# KOREAN VERSION OF PUBLIC STIGMA OF ALZHEIMER’S DISEASE SCALE: DEVELOPMENT AND EVALUATION AMONG KOREAN AMERICANS

**DOI:** 10.1093/geroni/igae098.1671

**Published:** 2024-12-31

**Authors:** Michin Hong, Sang Lee

**Affiliations:** Indiana University Indianapolis, Indianapolis, Indiana, United States; San Jose State University, San Jose, California, United States

## Abstract

This study aims to evaluate the psychometric properties of the Korean version of the public stigma of Alzheimer’s diseases scale (KPS-ADS). Stigma toward Alzheimer’s disease (AD) prevents the early diagnosis of AD, causing the delayed treatments. To our knowledge, there is no proper measure to assess AD public stigma among the Korean-speaking population. We used a community survey dataset with 268 Korean Americans. We translated the 19 items of the layperson’s stigma, a dimension of the Family Stigma in AD scale, using Brislin’s method. After randomly dividing the sample into two sub-groups, we conducted exploratory factor analysis (EFA) with one group to explore the factor structure of the KPS-ADS, and then performed confirmatory factor analysis (CFA) with the other group to validate its identified factor structure. Additionally, we performed reliability tests. EFA identified three factors: negative emotions, empathetic response, and social behaviors. Using this three-factor model, we performed CFA with the highest loading items loaded on each factor, but it showed a poor model fit. After removing three items, the revised model showed an excellent model fit (χ2 = 185.788, df = 97, p = 0.000, CFI = 0.955, TLI = 0.944, RMSEA = 0.081[90% CI: 0.063–0.099, p ≤ 0.05 = 0.003]). All loadings were significant. The KPS-ADS demonstrated excellent internal consistency reliability (α=.87), with each domain demonstrating excellent internal consistency. Our study presents a psychometrically sound, multifaceted KPS-ADS that contribute to better understanding the nature and magnitude of AD public stigma and reducing it in this population.

